# Association between video-based Pirani Böhm Sinclair score and treatment recommendations in recurrent clubfoot in walking-age children

**DOI:** 10.1186/s13018-026-07122-6

**Published:** 2026-07-24

**Authors:** Åsa Thelaus, Salik Kashif, Eva Broström, Alaric Aroojis, Steven Frick, Stephanie Böhm, Josefine Eriksson Naili

**Affiliations:** 1 Department of Women’s and Children’s Health, Karolinska Institutet, Karolinska vägen 37A, 171 76 Stockholm, Sweden; 2 Pediatric Orthopedic Dept, Karolinska University Hospital, Karolinska vägen 37A, 171 76 Stockholm, Sweden; 3 Department of orthopedic and spine surgery, Khyber girls Medical College, MTI Hayatabad Medical Complex Phase 4, Phase 5 Hayatabad, Pershawar, Pakistan; 4 Motion Analysis Lab, Karolinska University Hospital, Karolinska vägen 37A, 171 76 Stockholm, Sweden; 5 Lilavati Hospital, PD Hinduja Hospital, Bai Jerbai Wadia Hospital for Children, Mumbai, India; 6 Advocate Health Wake Forest University School of Medicine, 2001 Vail Ave, 6th Floor Musculoskeletal Institute, Charlotte, NC 28207 USA

**Keywords:** Congenital talipes equinovarus, Recurrence, Outcome, Function, Foot deformity

## Abstract

**Background:**

Management of recurrent clubfoot in walking-age children remains inconsistent, with substantial variation in treatment strategies. The role of clinical scoring systems in guiding treatment decisions in this group is unclear. This study investigated whether the video-based Pirani Böhm Sinclair (PBS)-score is associated with treatment recommendations.

**Methods:**

Fifty-five children aged 4–15 years (85 clubfeet) were video-documented. Four international paediatric orthopaedic surgeons independently assessed each case using the PBS-score and recommended management.

**Results:**

Higher total PBS-scores were significantly associated with lower odds of recommending observation (OR 0.2, *p* < 0.002). A threshold around PBS-score 10 marked a consistent shift from observation to active intervention. Median PBS-scores were 8 (7–10) for observation and 13 (11–16) for active treatment. Despite this association, there was substantial variability in the type of intervention recommended. Even when specific clinical findings, such as dynamic supination, were consistently identified, this did not uniformly translate into the same surgical decision.

**Conclusion:**

While higher PBS-scores are associated with the decision to intervene, considerable variation in treatment choice persists. These findings highlight the lack of consensus in managing recurrent clubfoot and underscore the need for clearer indications and standardized treatment algorithms.

*Trial registration* Clinical trial number NCT06050564, date of registration 2023-09-11.

**Supplementary Information:**

The online version contains supplementary material available at 10.1186/s13018-026-07122-6.

## Background

Idiopathic congenital talipes equinovarus (referred to as clubfoot from here on) is a relatively common congenital deformity, with an incidence of approximately 0.6–6.8 per 1000 live births and notable geographic variation [1]. Current evidence supports a multifactorial aetiology, with polygenic inheritance and environmental influences contributing to disease risk, although no single causative mechanism has been confirmed [1]. Family and epidemiological studies further suggest complex gene–environment interactions underlying the phenotype [1–4]. Recurrence of clubfoot deformity during childhood and adolescence has been reported to be ~ 11–48% [5, 6]. A recurrence does not resolve spontaneously. It needs to be identified early and treated to regain function and prevent worsening. The globally accepted initial treatment for newborn and infants with idiopathic clubfoot is the Ponseti method [7, 8]. Ponseti describes the main features of recurrence as reduced ankle dorsiflexion, heel varus, and increased lateral loading [9]. The treatment consisting of repeated serial casting and/or tenotomy of the Achilles tendon (TAL) depending on the type of recurrence [5, 10]. According to Ponseti, to prevent further recurrence, a transfer of the Tibialis anterior tendon (TATT) insertion from the first metatarsal and medial cuneiform to the lateral cuneiform can be performed in children aged over two and a half years [9]. This procedure reduces the deforming supinating forces to the foot [9, 11]. More extensive surgery such as posterior release is reserved for a few complicated cases with severe deformity where less invasive treatment such as serial casting, TAL and TATT is deemed futile.

Previous studies have shown large diversity among orthopaedic surgeons in the treatment of recurrence of deformity in walking children, as well as low level of agreement regarding definitions of recurrence and its identification [6, 12–14]. This is also reflected by the variety of available validated clinical scores used to assess functional status of clubfoot in walking children [15–18]. To increase consensus, an international Delphi process led to the publication of a core outcome set (COS) in 2022, containing a set of outcome measures for clinical practice and scientific reports on clubfoot [19]. The Pirani Böhm Sinclair (PBS)-score, included in the COS, is a validated score consisting of seven items that can be performed in a clinical setting [15]. The included items evaluate functional status of the foot with both static and dynamic elements. The total score ranges from 7 (a normal foot) to 18 (a foot with rigid deformity) [15].

In contrast to the general agreement and implementation of the Ponseti protocol in the newborn and infants with clubfoot there is no broad international consensus on how to best treat recurrence in walking children. Therefore, this study aimed to evaluate treatment recommendations, based on functional status assessed by video-documented PBS-score, of walking children with clubfoot. The treatment recommendations were given by senior paediatric orthopaedic surgeons representing three continents to gain a global perspective. Secondly, this study aimed to explore associations between specific items or combination of items included in the PBS-score and given treatment recommendations.

## Method

### Study design and study participants

This cross-sectional observational cohort study was conducted in accordance with the Helsinki declaration, and approved by the Swedish ethical review authority (Dnr 2022-03647-01) and the ethical review board of the Hayatabad Medical Complex, Pakistan (HMC-QAD-F-00 approval number 1210). The reporting of methodology and results was performed using the STROBE checklist [20]. Written informed consent to participate was provided from parents or legal guardian for all participating children. The study cohort consisting of 55 children age 4–15 years with a diagnosis of idiopathic clubfoot, recruited consecutively during clinical visits from two centres: Astrid Lindgrens Children’s Hospital, Karolinska University Hospital in Stockholm, Sweden, and Hayatabad Medical Complex, Orthopaedic Unit, Peshawar, Pakistan. Inclusion criteria included a diagnosis of idiopathic clubfoot, age 4–15 years, and ability to walk 10 m repeatedly. Exclusion criteria included syndromic- and/or neuromuscular clubfeet. The same cohort has previously been used in a study establishing the inter- and intra-rater reliability of video documented PBS-score (Table 1) [21].

**Table 1:** Demographics of the subjects included in the study and participating raters

Participating raters, *n*		4
Years of practice¹, median (range)		22 (7–26)
Male, n (%)		2 (50)
Country of residence, n	India	1
	USA	1
	Sweden	2
Study cohort, n		55
Bilateral, n (n of feet)		30 (60)
Unilateral		25
Sex male, n (%)		41 (75)
Age, in years, median (range)		7 (4–15)
PBS-score, median (range)		10 (7–18)

n, Number. ^1^ Years of practice as a paediatric orthopaedic surgeon

### Sample size estimation

Based on pilot data, a power analysis was conducted to compare the “need for treatment” group with the control group (no need for treatment) using the primary outcome measure (PBS-score). A between-group difference (delta) of two points was assumed, and a two-sided t-test with a significance level of α = 0.05 was applied. Power estimates were generated for sample sizes ranging from 40 to 100 participants, all of which yielded adequate power (87–99%). A target sample size of 50 participants was considered feasible and was expected to provide 94% power. All statistical analyses were performed using Stata version 15 (StataCorp, College Station, TX, USA).

### Rating protocol

A standardized rating protocol was developed by a subset of the authors (Josefine Eriksson Naili, Eva Broström, Stephanie Böhm, Salik Kashif, Åsa Thelaus) (S1). In the first section the raters scored each foot according to the PBS-score [15]. Results of inter-rater reliability and agreement have been published previously [21]. In the second section the raters provided a treatment recommendation, choosing between seven prefilled options. The raters were asked to choose ‘any that applied’ and were therefore able to choose as many options as they found appropriate. The prefilled alternatives included; Observe, Cast, Brace, TAL, TATT, Other soft tissue procedures, and Bony procedure. Options were ordered from left to right on the rating protocol in increasing invasiveness according to the authors (Josefine Eriksson Naili, Eva Broström, Stephanie Böhm, Salik Kashif, Åsa Thelaus) (S1). Additional space to add comments or remarks was provided.

### Participating raters

The group of raters consisted of four orthopaedic paediatric surgeons (Alaric Aroojis, Stephanie Böhm, Steven Frick, Åsa Thelaus) with median 22 (7–26) years of experience in treating clubfoot in clinical practice in Asia, North America and Europe, respectively (Table 1). Two of the raters had previous experience using the PBS-score. The two raters not familiar with the score, received the original publication on the PBS-score and the rating protocol beforehand.

### Rating session

**Fig. 1:**
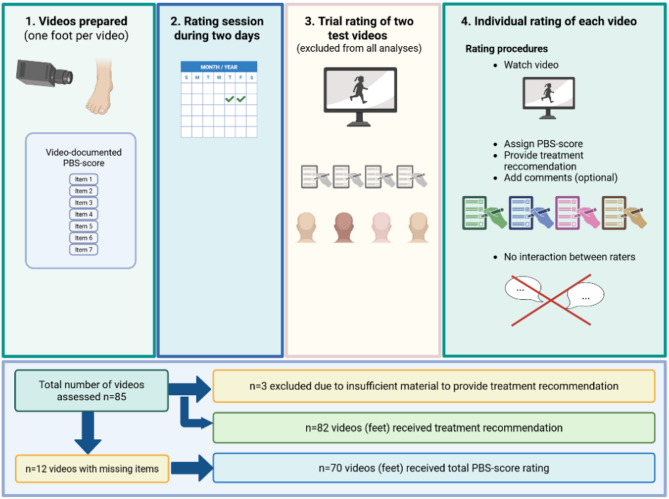
Overview of the rating procedure and video assessment process. Created in BioRender. Von Walden, F. (2026) https://BioRender.com/ifaiyxx

The rating procedure is illustrated in Fig. 1. Each foot was represented by one video containing all seven items of the PBS-score. Prior to watching each video, raters received information about age, sex and which foot (right or left) that should be assessed. All feet were reviewed in a standardized manner during a two-day live rating session. All raters initially reviewed and scored two feet to familiarize themselves with the procedure and protocol as a trial run. These two feet were not included in the analysis. After reviewing each foot and providing a PBS-score, the raters completed their treatment recommendation and any additional comments. No interaction between raters was allowed during rating.

### Recommended treatment

The recommended treatment for each foot was defined as the most invasive treatment chosen by each rater. Multiple treatment options were possible for each foot, reflecting a clinical setting where children with clubfoot recurrence often receive a combination of treatments. Therefore, if a foot was recommended for both Cast and TAL, it was identified as recommendation TAL in the analysis. Two separate categories, one combining conservative treatment (cast and brace) and one combining all surgical interventions was created to analyse agreement on treatment recommendations between raters.

### Agreement among raters

Agreement among raters was defined as when a recommended treatment was chosen by more than two raters i.e. if the treatment option was chosen by three or four of the raters (≥ 75%).

### Statistical analysis

Descriptive statistics were used to summarize population and rater characteristics, including means, minimum and maximum values, frequencies, and percentages. Each foot was treated as an independent observation in both unilateral and bilateral cases, and variables were analysed and reported separately for each foot. Linear regression was applied to evaluate the association between PBS-score and the probability of receiving the recommendation Observe [22]. In addition, a logistic regression model adjusted for age and PBS-score was used to calculate the odds ratio of being recommended Observe [23]. Further adjustments for foot affected (right/left), unilateral versus bilateral involvement, and sex did not improve the model. The recommended action for each participant was defined as agreement ≥ 75%. Possible actions included: Observe, Cast, Brace, TAL, TATT, Other soft tissue procedures, and Bony procedure. In addition, for the agreement analyses, Cast and Brace recommendations were combined into a conservative category, and all surgical procedures into a single surgical category. Two-tailed *p*-values < 0.05 were considered statistically significant. All statistical analyses were performed using Stata Statistical Software: Release 17 (StataCorp LLC, College Station, TX, USA; 2021).

## Results

**Fig. 2:**
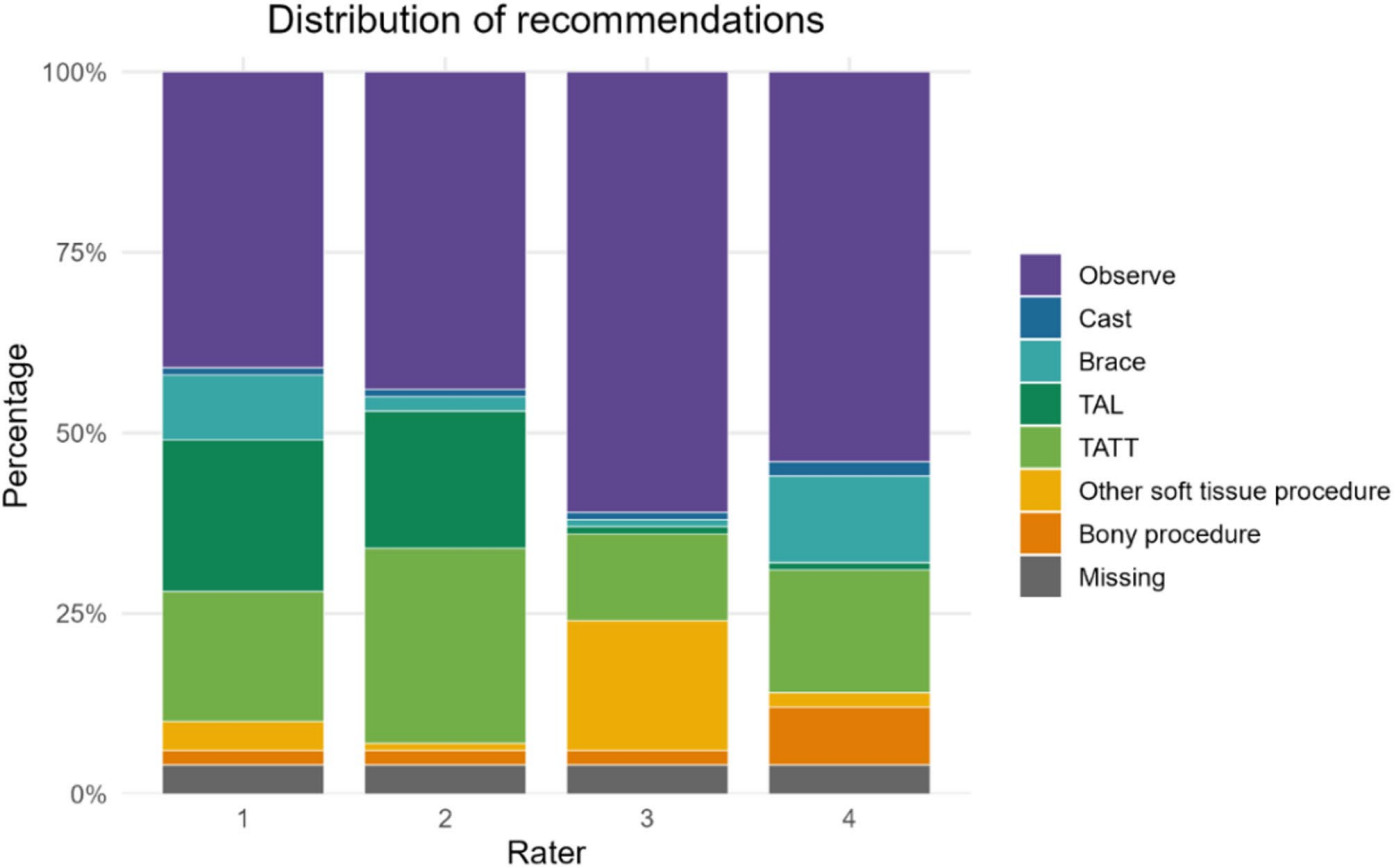
Distribution of recommended treatment across four raters. The bars illustrate the percentage distribution of recommended treatments for each rater. When multiple treatment options were selected for a given foot, the most invasive treatment was used for classification in the analysis. TAL, Tendo Achilles lengthening, TATT, Tibialis anterior tendon transfer

All four raters reviewed a total of 85 clubfeet, documented in a video containing the included items of the PBS-score for each foot respectively. The distribution of recommended treatment per rater is displayed in Fig. 2.

### Missing data

A total of three feet were excluded from the analysis due to not containing sufficient documentation of the PBS-score, thus lacking information for the rater to assess the foot. In 12 out of the remaining 82 feet, individual items of the PBS-score were missing, and a total score could not be calculated. Therefore 82 feet were included in the analysis of agreement and recommended treatment, while 70 feet were analysed in the linear and logistic regressions based on the total PBS-score (Fig. 1).

### Agreement on treatment recommendations

Observe was the only treatment recommendation with substantial agreement among raters. In 42 feet (51%) there was agreement on the recommendation of Observe (Table 2). For the recommendation of TATT there was agreement for 6 feet (8%). For any surgical intervention, all categories other than Observe, Cast, and Brace, there was agreement in 28 feet (33%) (Table 2).

### Total PBS-score and recommended treatment

**Fig. 3:**
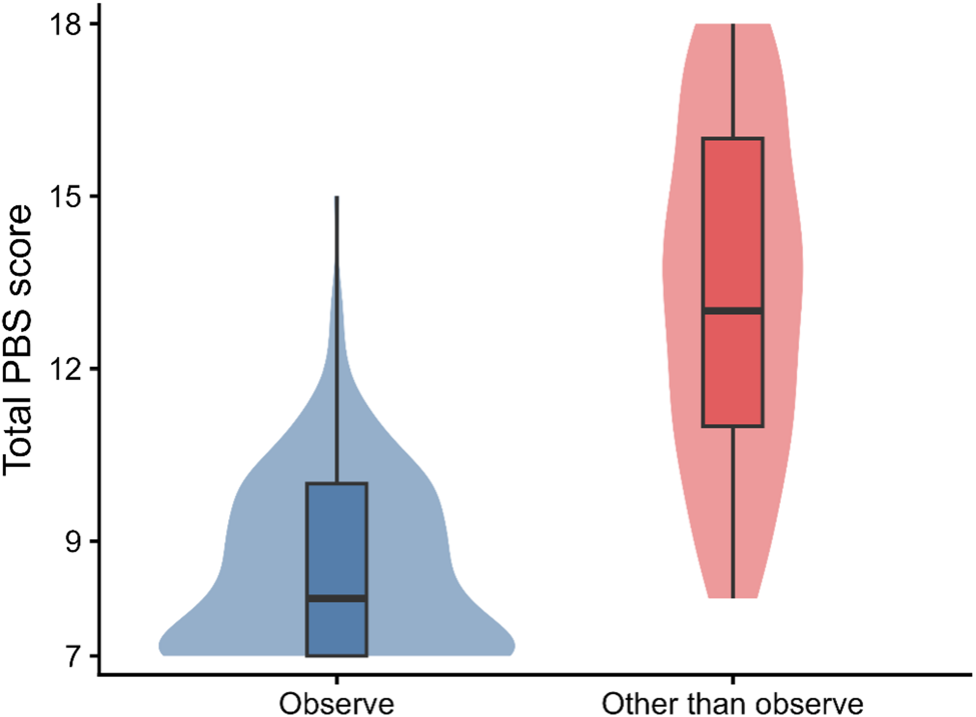
Total PBS-score distribution in individuals recommended for observe versus those receiving any type of treatment. Violin plot of the Pirani Böhm Sinclair (PBS)-score distribution in the group receiving the recommendation of observe and the group receiving any type of treatment recommended, respectively

**Fig. 4:**
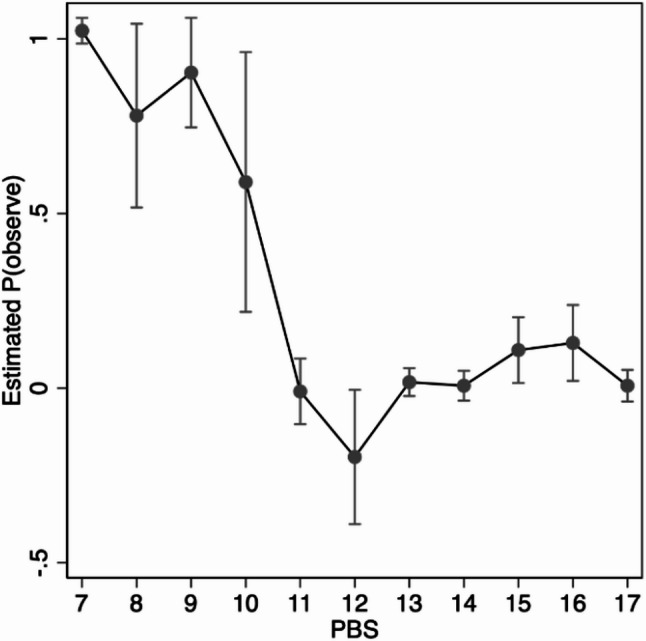
Odds of receiving recommendation of observe depending on total PBS-score. Linear regression of odds of recommendation being observe depending on the PBS-score

The median PBS-score in the group recommended Observe was 8 compared to 13 for any recommendation other than Observe (including cast, brace or any surgical intervention) (Fig. 3). There was a negative correlation between the total PBS-score and the odds of recommendations other than Observe (Fig. 4). The linear regression demonstrated a marked shift in the slope of the regression line and widening of the confidence interval at PBS-score 10. This indicates a threshold where the PBS-score identifies signs of recurrence and raters abandon the recommendation Observe in favour of active treatment, surgical or nonsurgical. (Fig. 4). Further, in the logistic regression model adjusting for age, an increase of 1 unit in the PBS-score was associated with a 0.2 odds ratio of the recommendation Observe (*p* = < 0.01). For each point the PBS-score increases, the odds of receiving the recommendation Observe is five times less (Table 3).

**Table 2:** Agreement on recommended treatment between raters in the total cohort, and treatment recommendations for each rater respectively

Recommended treatment	No agreement voted by < = 2 raters *n* (%)	Agreement voted by > 2 raters *n* (%)	Rater 1	Rater 2	Rater 3	Rater 4
Observe	40 (49)	42 (51)	35(41)	37 (44)	52 (61)	46 (54)
Cast	82 (100)	–	1 (1)	1 (1)	1 (1)	2 (2)
Brace	82 (100)	–	8 (9)	2 (2)	1 (1)	10 (12)
TAL	82 (100)	–	18 (21)	16(19)	1 (1)	1 (1)
TATT	76 (92)	6 (8)	15 (18)	23(27)	10 (12)	14 (17)
Other soft tissue	82 (100)	–	3 (4)	1 (1)	15 (18)	2 (2)
Bony procedure	81 (99)	1(1)	2 (2)	2 (2)	2 (2)	7 (8)
Cast or Brace	82 (100)	–	9 (11)	3 (4)	2 (2)	12 (14)
All surgical interventions	54 (66)	28 (34)	38(45)	42(49)	28 (33)	24 (28)
Missing			3 (4)	3 (4)	3 (4)	3 (4)

**Table 3:** Logistic regression of association between PBS-score and recommended action observe

Action observe	Odds ratio	Robust standard- error	z	*P *> ı z ı	95% confidence-interval
PBS	0.2	0.103	− 3.12	< 0.01	0.07 0.05

### Individual items and recommended treatment

**Fig. 5:**
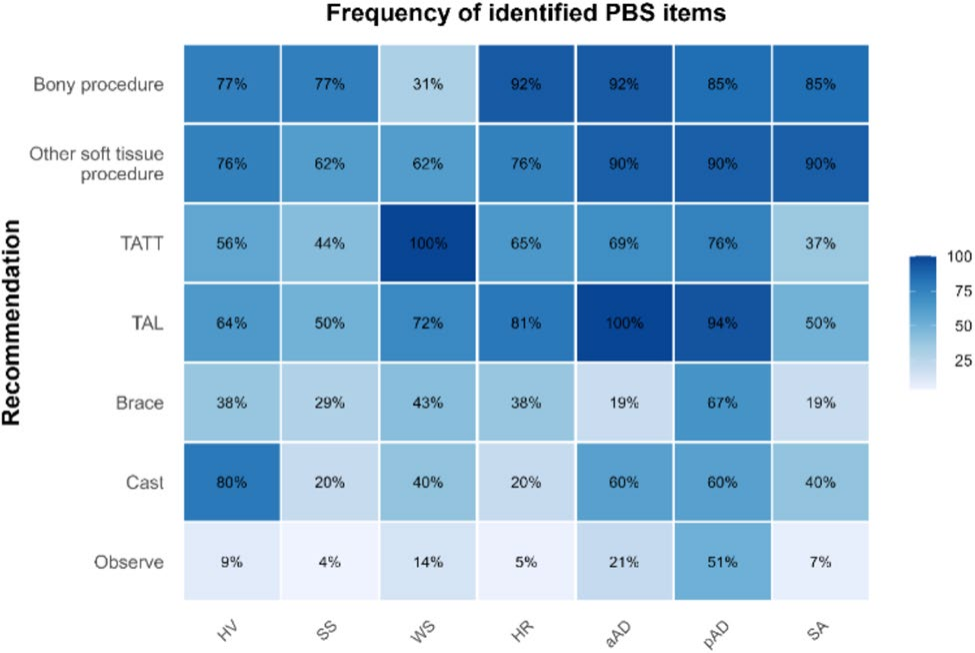
Identified items of PBS-score in relation to recommended treatment. Heatmap of frequency of Pirani Böhm Sinclair (PBS)-score individual items scored as present or reduced in relation to the recommended treatment. TATT, Tibialis anterior tendon transfer; TAL, Tendo Achilles lengthening; HV, hindfoot varus; SS, standing supination; WS, walking supination; HR, early heel rise; aAD, active ankle dorsiflexion; pAD, passive ankle dorsiflexion; SA, subtalar abduction

In all feet recommended TATT, all four raters also identified the item ‘walking supination’ (WS) as present (Fig. 5). Concurrently, all feet recommended for TAL had the item ‘active ankle dorsiflexion’ (aAd) identified by all raters respectively (Fig. 5), i.e. reduced range of motion in aAd. However, not all feet that were identified as presenting with WS were recommended a TATT.

### Comments and additional remarks

A complete list of additional free text comments is disclosed as supplementary material (S4). Notably, rater 2 recommended physiotherapy for several subjects, a treatment not included in the preset options. Rater 3 made remarks about requiring x-ray of the foot and ankle to be able to make a definite treatment recommendation. Plantar fascia release (PFR) was the most frequent definition of what constitute other soft tissue procedure.

## Discussion

### Agreement on treatment recommendation

In this study, agreement on treatment recommendations among raters was low. Regarding feet that did not need treatment and feet needing any type of surgical treatment, there was agreement to some extent (Do nothing/ Observe 49% and surgical treatment 33%, respectively). In a review article from 2017, Hosseinzadeh et al. described treatment strategies for clubfoot recurrence in line with the Ponseti protocol, emphasizing the importance of manipulation and cast treatment in all recurrences, and also preoperatively [13]. In the review, other surgical procedures, not included in the Ponseti protocol, are described. In 2019, Morcuende et al. published a survey performed in the Paediatric Orthopedic Association of North America (POSNA) about treatment of the recurrent clubfoot [12]. Although initial treatment was performed according to Ponseti principles, there was little agreement on how to identify and treat recurrence. Notably only 62% of respondents used a preoperative cast before performing a TATT, and 43% reported performing a posterior capsule release in concordance with TATT, both of which deviate from the treatment principles of the Ponseti method [12].

In a more recent British multicentre study by Gelfer et al. from 2022, 35% of recurrences received re-casting, re-TAL and TATT while soft tissue releases and osteotomies were reported in only 5% and 2%, respectively [6]. In the present study, two of the four raters recommended preoperative cast to all (100%) patients recommended for any type of surgery. The other two raters made the same recommendation in 97% and 71% of the patients, respectively (S3). In comparison to the POSNA survey and Gelfer et al., the raters as a group in our study recommended preoperative cast in 90% of all patients receiving a recommendation of surgical treatment. Cast as the only intervention was only recommended in 1–2% in the present study. According to the Ponseti protocol resumed casting is always the first step, and sometimes the only intervention needed in treating recurrence [9]. However, predicting the outcome of resumed serial cast as the only intervention is difficult due to varying responsiveness to the treatment between feet [24]. Thus, a recommendation involving surgery might be abandoned after achieving a good correction of the foot with the preoperative cast only and a foot that does not achieve sufficient correction with serial casting might need surgery although this was not a part of the original recommendation. One should also bear in mind that treatment strategies for recurrence are not determined solely by severity of deformity. Personal adherence, socioeconomic conditions, and access to care critically influence whether repeat Ponseti casting is feasible [25].

### Total PBS-score and recommended treatment

In a previous study, it was established that the total PBS-score based on video documented material has high inter- and intra-rater reliability [21]. Results from the present study highlight that the treatment recommendation given is dependent on the total PBS-score. When comparing children, a foot that received the recommendation Observe had an average PBS-score that was 5 points lower than a foot that was recommended to any type of treatment (Fig. 3). The median PBS-score in the group of children recommended to receive treatment was 13. To receive PBS-score 13, more than two (sometimes four) of the included seven items must be scored as deviant. This clearly separates a child with a recurrence from a child with a well corrected foot when using the total PBS-score for treatment decision making. Further, results of the present study identified a threshold at PBS-score 10 when raters abandon Observe in favour of initiating treatment (Fig. 4). Possibly indicative of a level where the PBS-score identifies early signs of recurrence. However, results of the current study could not establish any agreement among raters regarding what treatment is recommended for each individual foot. This finding points out the need for further studies on the indications for treatment of clubfoot recurrence after initial Ponseti method correction, and then development of a decision-tree to facilitate assessment and the best treatment for recurrent deformity.

### Treatment recommendation in relation to individual items of PBS-score

Dynamic supination during swing phase of gait, at any age, seems to be a clinically important sign for surgeons in deciding for a TATT [11, 26]. However, half of the raters in our study (2/4) also scored standing supination (SS) as present in 53% and 65% of individuals with WS respectively, indicating a fixed deformity in the children recommended for TATT. The definition of WS in relation to SS has been discussed in a previous study on inter- and intra-rater reliability [21]. A clarification whether SS and WS can be present simultaneously is needed to improve validity and reliability of the PBS-score. If there is SS in addition to WS, then the rigid deformity needs to be corrected prior to TATT, as a principle of tendon transfers is that they will maintain but not obtain correction of rigid deformities [27].

## Limitations

The power calculation for sample size estimation was based on the difference between a “need for treatment” group and a control group where the difference was assumed to be two points on the PBS-score. Thus, for the logistic regression models the current sample size may be insufficient. Readers are therefore referred to the confidence intervals for cautious interpretation. The fair and moderate levels of inter-rater reliability of individual items of video documented PBS-score are inherited and as such affects the results of the present study.

## Conclusion

This study shows there is marked variation in treatment recommendations for recurrent clubfoot after initial Ponseti method treatment by experienced, international surgeons with clubfoot expertise. While higher PBS-scores are associated with the decision to intervene, considerable variation in treatment choice persists. Further studies are needed to define the indications for treatment of recurrent clubfoot, and to clarify the best treatment methods for common recurrent clubfoot deformity such as cavus, equinus, equinovarus, varus, or dynamic supination of the fore foot during gait.

## Supplementary Information

Below is the link to the electronic supplementary material.

Supplementary Material 1

Supplementary Material 2

Supplementary Material 3

## Data Availability

The datasets generated during the current study are not publicly available due to containing personal information but are available from the corresponding author on reasonable request.

## Abbreviations

aAD: Active ankle dorsiflexion
COS: Core outcome set
HR: Early heel rise
HV: Hindfoot varus
pAD: Passive ankle dorsiflexion
PBS-score: Pirani Böhm Sinclair score
PFR: Plantar fascia release
SA: Subtalar abduction
SS: Standing supination
TAL: Tendo achilles lengthening
TATT: Tibialis anterior tendon transfer
WS: Walking supination
