# Supplementary material for: Association between video-based Pirani Böhm Sinclair score and treatment recommendations in recurrent clubfoot in walking-age children
Source: J Orthop Surg Res. 2026 Jul 24;21:443. doi: 10.1186/s13018-026-07122-6 (PMC13397732; doi:10.1186/s13018-026-07122-6)
Supplement: Supplementary file 1 — Supplementary Material 1 [file 13018_2026_7122_MOESM1_ESM.docx]

| Procedure type | Total | Rater 1 | Rater 2 | Rater 3 | Rater 4 |
| --- | --- | --- | --- | --- | --- |
| Any type of surgery | 119/132 (90) | 37/38 (97) | 30/42 (71) | 28/28 (100) | 24/24 (100) |
| TAL, n (%) | 31/36 (86) | 17/18 (94) | 12/16 (75) | 1/1 (100) | 1/1 (100) |
| TATT, n (%) | 54/62 (87) | 15/15 (100) | 15/23 (65) | 10/10 (100) | 14/14 (100) |
| Other soft tissue procedure, n (%) | 21/21 (100) | 3/3 (100) | 1/1 (100) | 15/15 (100) | 2/2 (100) |
| Bony procedure, n (%) | 13/13 (100) | 2/2 (100) | 2/2 (100) | 2/2 (100) | 7/7 (100) |

*Supplement 3. Number of feet prescribed a preoperative cast in total and for each rater respectively*.
