# Supplementary material for: Association between video-based Pirani Böhm Sinclair score and treatment recommendations in recurrent clubfoot in walking-age children
Source: J Orthop Surg Res. 2026 Jul 24;21:443. doi: 10.1186/s13018-026-07122-6 (PMC13397732; doi:10.1186/s13018-026-07122-6)
Supplement: Supplementary file 2 — Supplementary Material 2 [file 13018_2026_7122_MOESM2_ESM.docx]

| *Supplement 2.* *Free text comments per foot and rater respectively.* | | | | | |
| --- | --- | --- | --- | --- | --- |
| **Study id** | **Free text Rater 1** | **Free text Rater 2** | **Free text Rater 3** | **Free text Rater 4** | |
| 1 |  |  | mild fft varus/flexible | Physio | |
| 2 |  |  |  |  | |
| 3 |  |  |  |  | |
| 4 | likely TA lengthening and ATT and Bracing |  | PFR | potential PFR | |
| 5 |  |  | PFR | potential PFR | |
| 6 |  | calf stretch | Prominent P-L calc |  | |
| 7 |  |  |  | evaluate after pre op cast | |
| 8 |  |  | assess ankle DF intra-op, if <15 do TA | continue FAB after cast | |
| 9 |  | active eversion exercises | some forefoot supination but does not have ic with lateral border |  |  |
| 10 |  |  |  |  | |
| 11 |  |  |  |  | |
| 12 |  |  |  |  | |
| 13 | ATL depending on intra op movement |  | if tripping/symptomatic cuboid/cuneiform osteotomy. Assess for excess internal tibial torsion | evaluate after cast, prob calcaneal shift | |
| 14 | ATL depending on intra op movement |  |  | If tripping/symptomatic-PMR/TAL, cuboid/cuneiform osteotomy. | |
| 15 |  |  |  |  | |
| 16 |  | calf stretches |  |  | |
| 17 | Maybe bony procedure (equinus) in the future | peroneal activation exercise |  | for drop foot | |
| 18 | Maybe bony procedure (equinus) in the future | peroneal activation exercise | consider external rotation osteotomy rt distal tibia |  |  |
| **Study id** | **Free text Rater 1** | **Free text Rater 2** | **Free text Rater 3** | **Free text Rater 4** | |
| 19 |  |  |  |  | |
| 20 |  | peroneal activation exercise |  |  | |
| 21 | Due to no pain and no limitation | active calf stretch/ peroneal activation exercise |  |  |  |
| 22 | Due to no pain and no limitation | active calf stretch/ peroneal activation exercise |  |  |  |
| 23 |  |  | PFR- prior TAL |  | |
| 24 |  |  | PFR |  | |
| 25 |  | active calf stretches |  |  | |
| 26 |  | active calf stretches |  | TAL depending on ROM intra-Op | |
| 27 |  |  | PFR |  | |
| 28 |  |  | PFR |  | |
| 29 |  |  |  |  | |
| 30 |  |  |  |  | |
| 31 |  |  |  |  | |
| 32 |  | may need TATT, may need lateral column shortening | PFR, cuboid, LCS | stepwise posterior release, calcaneal shift and midfoot osteotomy | |
| 33 | No brace due to age | active calf stretches |  |  | |
| 34 | No brace due to age | active calf stretches |  |  | |
| 35 |  |  |  |  | |
| 36 |  |  | Return 3months, at risk |  | |
| 37 |  |  |  |  | |
| 38 |  | Active peroneal exercise |  |  | |
| 39 |  | May need ATT later |  |  | |
| 40 |  | May need ATT later |  |  | |
| 41 |  | Active calf stretches |  |  | |
| 42 |  |  |  |  | |
| **Study id** | **Free text Rater 1** | **Free text Rater 2** | **Free text Rater 3** | **Free text Rater 4** |  |
| 43 | Bracing after ATT |  | cast pre op, lengthen tib post and ta/posterior release |  | |
| 44 |  | Will need ATT later | PFR,TAL and PMR | midfoot, calcaneus and possibly tibia | |
| 45 |  | May need plantar fascia release | cast pre op, PFR, Cuboid osteotomy, tib post lengthening, tn medial capsulotomy, TAL | midfoot, calcaneus and possibly tibia | |
| 46 |  | Active calf stretches | in-between mild dyn sup, might observe | possibly TA during surgery | |
| 47 |  |  |  |  | |
| 48 | Bracing post ATT |  |  |  | |
| 49 | ATT in case of incompliance or long distance to travel | May need ATT later | PFR, possible LCS |  | |
| 50 |  | Active calf stretches |  |  | |
| 51 |  | Active calf stretches |  |  | |
| 52 |  |  |  |  | |
| 53 | Maybe bracing after TAL for a short period of time | Active calf stretches | X-ray would help me decide limited ankle DF, plantar callus only on forefoot |  |  |
| 54 | Bracing after ATT for a limited period of time | May need ATT later | PFR, possible LCS/cuboid osteotomy |  | |
| 55 | Bracing after ATT for a limited period of time | May need ATT later |  |  | |
| 56 |  | Active calf stretches, peroneal activation exercise | early heel rise one step |  | |
| 57 |  | Active calf stretches | HF equinus, MF DF x-ray would show | physio | |
| 58 |  |  | cavus - PFR |  | |
| 59 |  |  | PFR |  | |
| 60 | Bracing after ATT for a limited period of time | May need ATT later |  |  | |
| 61 | Bracing after ATT for a limited period of time | May need ATT later | cavus? Possible PFR |  | |
| **Study id** | **Free text Rater 1** | **Free text Rater 2** | **Free text Rater 3** | **Free text Rater 4** | |
| 62 |  | Peroneal activation exercise/May need ATT later |  |  |  |
| 63 | 8plates | May need lateral collum shortening osteotomy | PMR/TAL/PFR/LCS-CO | prob 1st MT and calcaneus dep on talus flat? | |
| 64 | 8plates | Active calf stretches |  |  | |
| 65 | Bracing after ATT for a limited period of time | Active calf stretches/ May need ATT later |  |  | |
| 66 | up to 5 |  |  |  | |
| 67 | 1 or 2 casts and continue brace to age 5 | Active calf stretches |  |  | |
| 68 |  |  | Possible PFR |  | |
| 69 |  | Active calf stretches |  |  | |
| 70 |  | Active calf stretches |  |  | |
| 71 | if not effective ATT and re-tenotomy and brace | 1-2 cast followed by ATT |  |  | |
| 72 | later ATT and TAL if necessary | 1-2 cast followed by ATT |  | continue FAB at night | |
| 73 | later ATT and TAL if necessary | 1-2 cast followed by ATT |  |  | |
| 74 |  | Active calf stretches/ May need ATT later |  |  | |
| 75 |  | Active calf stretches/ May need ATT later |  |  | |
| 76 | and observe hind foot varus | Active calf stretches | could continue brace till 5 | continue FAB | |
| 77 |  |  |  |  | |
| 78 |  |  | could brace till 5 | continue FAB to 5 | |
| 79 |  |  |  |  | |
| 80 |  |  |  |  | |
| 81 |  |  |  |  | |
| 82 |  |  |  |  | |
| 83 |  |  |  |  | |
| 84 |  | Active calf stretches |  |  | |
| **Study Id** | **Free text Rater 1** | **Free text Rater 2** | **Free text Rater 3** | **Free text Rater 4** | |
| 85 |  | Active calf stretches |  |  | |
