# Supplementary material for: Association between video-based Pirani Böhm Sinclair score and treatment recommendations in recurrent clubfoot in walking-age children
Source: J Orthop Surg Res. 2026 Jul 24;21:443. doi: 10.1186/s13018-026-07122-6 (PMC13397732; doi:10.1186/s13018-026-07122-6)
Supplement: Supplementary file 3 — Supplementary Material 3 [file 13018_2026_7122_MOESM3_ESM.docx]

**Supplement 1**

Patient ID _____________________________________

Examiner name ________________________________

**What would you recommend for this patient?**

Circle any that apply.

| **Left foot** | Do nothing/ observe | Cast | Bracing | Lengthen Achilles tendon | Anterior Tendon Transfer | Other soft tissue procedure | Bony procedure |
| --- | --- | --- | --- | --- | --- | --- | --- |

Other: ________________________________________________________________

| **Right foot** | Do nothing/ observe | Cast | Bracing | Lengthen Achilles tendon | Anterior Tendon Transfer | Other soft tissue procedure | Bony procedure |
| --- | --- | --- | --- | --- | --- | --- | --- |

Other: ________________________________________________________________

Additional comments/remarks: _____________________________________________________________________

_____________________________________________________________________

_____________________________________________________________________
